# Newborn Screening for Cystic Fibrosis: Infant and Laboratory Factors Affecting Successful Sweat Test Completion

**DOI:** 10.3390/ijns7010001

**Published:** 2020-12-25

**Authors:** Ambika Shenoy, Dina Spyropoulos, Kathleen Peeke, Dawn Smith, Michael Cellucci, Aaron Chidekel

**Affiliations:** 1 Division of Pulmonology, Nemours, Alfred I. duPont Hospital for Children, 1600 Rockland Road, Wilmington, DE 19803, USA; dinaspyro@gmail.com (D.S.); kathleen.peeke@nemours.org (K.P.); achidek@nemours.org (A.C.); 2 Division of Laboratory Medicine, Nemours, Alfred I. duPont Hospital for Children, 1600 Rockland Road, Wilmington, DE 19803, USA; dawn.smith@nemours.org; 3 State of Delaware Newborn Screening Program, 1600 Rockland Road, Wilmington, DE 19803, USA; michael.cellucci@nemours.org

**Keywords:** newborn screening, neonatal screening, cystic fibrosis, sweat chloride testing, immune reactive trypsinogen

## Abstract

Newborn screening (NBS) for Cystic Fibrosis (CF) has revolutionized the diagnosis of this inherited disease. CF NBS goals are to identify, diagnose, and initiate early CF treatment to attain better health outcomes. Abnormal CF NBS infants require diagnostic analysis via sweat chloride testing (ST). During ST, insufficient sweat volume collection causes a “quantity not sufficient” (QNS) test result and may delay CF diagnosis. The CF Foundation recommends QNS rates <10% for infants <3 months, but many CF Centers experience difficulties meeting this standard. Our quality improvement (QI) study assessed infant and laboratory factors contributing to ST success and QNS rates from 2017–2019. Infants’ day of life (DOL) at successful ST completion was analyzed according to infant factors (birth weight (BW), gestational age, ethnicity, and sex). Laboratory factors and procedures affecting ST outcomes were also reviewed. At our institution, BW and gestational age were the infant factors found to significantly affect DOL at ST completion. ST education, reduced number of laboratory technicians, and direct observation during ST completion also improved ST success rates. This study supports QI measures and partnerships between CF centers and laboratory staff to identify and improve ST QNS rates while sustaining practices to ensure timely CF diagnostic testing.

## 1. Introduction

Cystic fibrosis (CF) is a life-threatening, genetic condition affecting approximately 1/3500 births annually in the United States [1]. Newborn screening (NBS) for CF has revolutionized the diagnosis and early management of this common, inherited disease [2]. The goal of CF NBS is to achieve early CF diagnosis so that comprehensive medical and psychosocial therapies can be implemented in infants prior to the onset of clinical symptoms to ensure better disease outcomes [3,4,5,6,7,8]. Despite mandated CF NBS in 2010 across the United States, in 2018 only 61.5% of newly diagnosed CF patients were identified through NBS [9]. The European Cystic Fibrosis Society Patient Registry Report in 2018 similarly stated that 74% of CF patients aged 5 years had undergone CF NBS at birth (https://www.ecfs.eu/projects/ecfs-patient-registry/annual-reports). Taken together, these data support additional quality improvement (QI) in CF NBS to better understand the current screening practices in place, identify factors contributing to delays in diagnostic testing and identify barriers to receiving specialized CF multidisciplinary, clinical, and psychosocial care.

As a screening test, CF NBS meets all of the requirements in terms of its sensitivity, specificity and positive/negative predictive value which varies depending on the NBS protocol employed by each program [10]. Elevated serum immunoreactive trypsinogen (IRT), a protein marker of pancreatic inflammation/disease, is the first indicator of positive screening which triggers a second tier of testing [11,12]. Utilization of DNA testing in combination with IRT improves sensitivity and specificity of diagnosing patients identified with abnormal NBS with CF [13].

All infants with an abnormal or inconclusive CF NBS require diagnostic sweat chloride testing (ST), the gold standard test for diagnosing CF [14]. ST assesses Cystic Fibrosis Transmembrane Conductance Regulator (CFTR) Protein function in the sweat glands of the skin [15]. ST values ≥60 mmol/L are consistent with CF and require repetition on a second day for confirmation [16]. ST values <30 mmol/L make the possibility of CF unlikely. ST values between 31–59 mmol/L are indeterminate and require repeat [16] and possibly additional testing [17]. The Cystic Fibrosis Foundation (CFF) requires ST on all infants with CF as a criterion for inclusion in the CF Patient Registry in the United States [16].

ST, stimulated by pilocarpine iontophoresis, is collected via Gibson Cook Technique or Macroduct Sweat Collection System (MSC) (Wescor Inc. Logan, UT, USA). It is imperative that ST be completed in CFF and CLABSI-accredited laboratories with protocols in place to ensure quality control and reliable test results [18,19]. Insufficient yield of sweat volume is called ‘quantity not sufficient’ (QNS), and may prolong CF diagnosis and initiation of appropriate therapy. The CFF recommends QNS rates ≤10% for infants <3 months of age. As younger infants are now commonly referred for diagnostic testing following identification through abnormal CF NBS results rather than onset of CF symptoms, this can be a difficult goal to meet [20,21] and provides the rationale for CF centers and institutions to assess their screening and diagnostic programs. As infant and laboratory factors may contribute to ST QNS rates, a standardized approach is needed to review various contributing elements so that they may be addressed to subsequently reduce QNS occurrences.

The Nemours-A.I. duPont Hospital for Children is an accredited CF Center that routinely assesses infants with abnormal CF NBS from Delaware and surrounding States including New Jersey and Pennsylvania. In this QI study, QNS rates were assessed and the contribution of various infant and laboratory factors were examined in a systematic manner in order to identify areas for enhancement, reduce QNS rates, and improve time to CF diagnosis.

## 2. Materials and Methods

Data were gathered through an IRB-reviewed and exempted chart analysis that queried electronic medical records of infants referred and evaluated for abnormal CF NBS from January 2017–December 2019. A deidentified CF NBS database in Microsoft Excel was used to store and analyze data. Infants were identified with abnormal CF NBS based on individual State NBS testing strategy. Delaware transitioned from IRT/IRT/DNA to IRT/DNA testing on 1 January 2018. The IRT/DNA strategy is equivalent to that utilized by the State of Pennsylvania. In both States, infants with IRT >96% of all values for the day undergo reflex 39+4 CFTR gene mutation analysis with any CFTR mutations identified included in the report. The State of New Jersey also utilizes IRT/DNA testing strategy, and infants with elevated IRT undergo CFTR gene mutation analysis for p.Phe508del mutation. Either the State NBS program or Primary Care Provider (PCP) notifies the CF NBS clinic of abnormal test results so that the family can be contacted to schedule diagnostic ST. Preterm infants, or those requiring inpatient hospitalization were scheduled for ST provided that they were tolerating an enteral diet and were stable off supplemental oxygen, often following hospital discharge.

ST was completed in the Nemours Department of Clinical Laboratory Medicine per manufacturer and CFF guidelines [18,22]. Using the MSC system, ST was performed in duplicate collections from the infant’s forearms or thighs. QNS was defined as <15 mL of sweat production in a 30 min period. As part of QI measures at our Institution, the CF NBS clinical team (MD, APN) met routinely with clinical laboratory leadership to review ST success and QNS rates. Initially these meetings occurred semi-annually. Based on QNS rate fluctuations, quarterly meetings were initiated in June 2018 to review infant factors and laboratory factors. After noting QNS rates decreased immediately following scheduled quarterly meetings, ST review meetings were increased to monthly in December 2018.

Additionally, direct observation of laboratory personnel completing ST was initiated in July 2018 in combination with education provided by the MSC/PorterCreek representative. A laboratory consultant from MSC provided additional education in January 2019 and again in May 2019. Throughout the entire timeframe of study, Nemours laboratory staff ensured that MSC equipment was functioning appropriately, reagents were not outdated or expired, and no single clinical laboratory specialist performing ST had a disproportionate number of QNS tests compared with their peers. Prior QI recommendations suggest direct observation and limiting laboratory testing to a smaller number of well-trained individuals that can complete at least 1 ST per week improves QNS rates [20]. Direct observation of ST completion by clinical laboratory leadership was routinely instituted at our center in May 2019. Limiting the number of personnel completing ST from 5 to 2 individuals in July 2019 and also reducing the number of days that ST was available was also helpful in reducing QNS rates. Table 1 summarizes QI initiatives pursued to decrease ST QNS rates.

When scheduling ST appointments, families were routinely instructed on the importance of infant hydration and avoidance of emollients which may interfere with sweat production during a pre-ST appointment telephone encounter with the CF NBS clinic coordinator. In July 2019, a second reminder the night prior to ST completion was initiated and provided to families by clinical laboratory staff.

Infants’ day of life (DOL) at successful ST completion was analyzed according to birth weight (BW), gestational age, sex, ethnicity, presence of skin lesions. When analyzing BW, infants were stratified by weight: <2.0 kg, 2.0–3.0 kg, 3.0–3.5 kg, and >3.5 kg. When analyzing gestational age, infants were stratified as preterm (gestational age <37 weeks) or term (gestation age ≥37 weeks). Infants were also stratified by ethnicity: Caucasian, African American, Hispanic, Asian, or Other (mixed ancestry or data not provided by parent/screening State). The data are expressed as median DOL at ST completion (in days), DOL range from minimum to maximum (in days). Nonparametric analyses were used to assess for statistically significant differences using R software. Given multiple subgroups for BW and ethnicity, a Kruskal Wallis test was performed to compare values. When significant differences were noted, post hoc Dunn testing was completed to note differences between paired subgroups. A Mann Whitney U test was used to compare median values for sex and gestational age. A *p* < 0.05 was considered significant. CF infants who were diagnosed based on CFTR gene analysis and those who completed diagnostic testing at other CF Centers were excluded from DOL at ST completion analysis as data were not available for review.

Infants with QNS ST results were rescheduled for repeat ST approximately 7–14 days following initial testing. PCPs and families were updated with test results and were counseled on signs and symptoms of CF. They were advised to call in the interim should any symptoms manifest while awaiting diagnostic ST. Psychosocial support was also available if needed. Fecal elastase testing was suggested for infants with concern for malnutrition or steatorrhea. In January 2019, infants were scheduled for ST around estimated weight of 3.0 kg following initial review of infant factors contributing to ST QNS rates.

## 3. Results

Overall, 160 infants with abnormal CF NBS were referred between January 2017 through December 2019 for additional diagnostic testing. Table 2 shows the number of infants referred from Delaware and surrounding States. As shown, the majority of patients were referred from the State of Delaware (*n* = 111 infants), followed by Pennsylvania (*n* = 42 infants), and lastly New Jersey (*n* = 7 infants). Infants encompassed varying ethnic backgrounds. Outcomes of infants referred with abnormal CF NBS are shown in Table 2. A number of infants were identified as CF Carriers (*n =* 54 infants) with 1 CFTR mutation reported on State CF NBS report and ST results <30 mmol/L. Infant factors were examined in infants successfully completing ST within the first 6 months of life who were referred due to abnormal CF NBS.

### 3.1. Infant Factors

#### 3.1.1. Birth Weight

**Figure 1:**
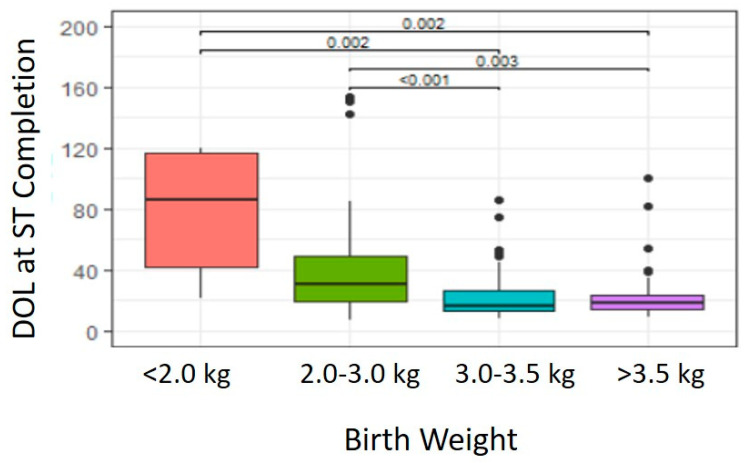
Effect of Birth Weight on Age at Successful Completion of Sweat Chloride Testing.

As shown in Figure 1, significant differences were noted in DOL at ST completion based on BW (*p* < 0.001). Post-hoc analysis of paired subgroups indicated a significantly earlier DOL at ST completion in infants with BW 3.0–3.5 kg (Median 16 days, Range 8–86 days, *n* = 58 infants) compared with infants whose BW was 2.0–3.0 kg (Median 31 days, Range 7–154 days, *n =* 49 infants) (*p* < 0.001) or <2.0 kg (Median 85.5 days, Range 21–120 days, *n =* 6 infants) (*p =* 0.002). Similarly, infants with BW >3.5 kg (Median 18 days, Range 9–100 days, *n* = 45 infants) also exhibited earlier DOL at ST completion compared with infants whose BW was 2.0–3.0 kg (*p =* 0.003) or <2.0 kg (*p* = 0.002). No significant differences were noted in DOL at ST completion for infants with BW 3.0–3.5 kg compared with those whose BW >3.5 kg (*p =* 0.701) or infants with BW <2.0 kg compared with those whose BW ranged 2.0–3.0 kg (*p =* 0.155).

#### 3.1.2. Sex

**Figure 2:**
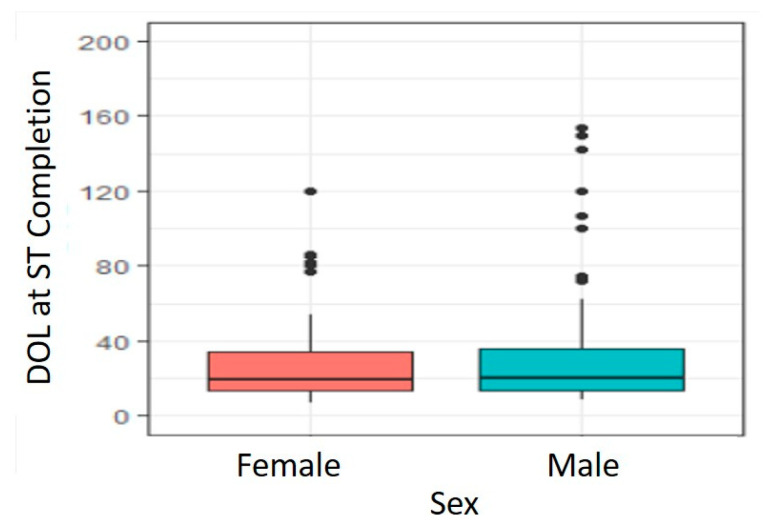
Effect of Sex on Age at Successful Completion of Sweat Chloride Testing.

There was no significant difference in DOL at ST completion due to sex (*p =* 0.468). As shown in Figure 2, male infants DOL at successful ST completion (Median 20 days, Range 9–154 days, *n =* 69 infants) was similar to female infants (Median 19 days, Range 7–120 days, *n =* 85 infants).

#### 3.1.3. Ethnicity

**Figure 3:**
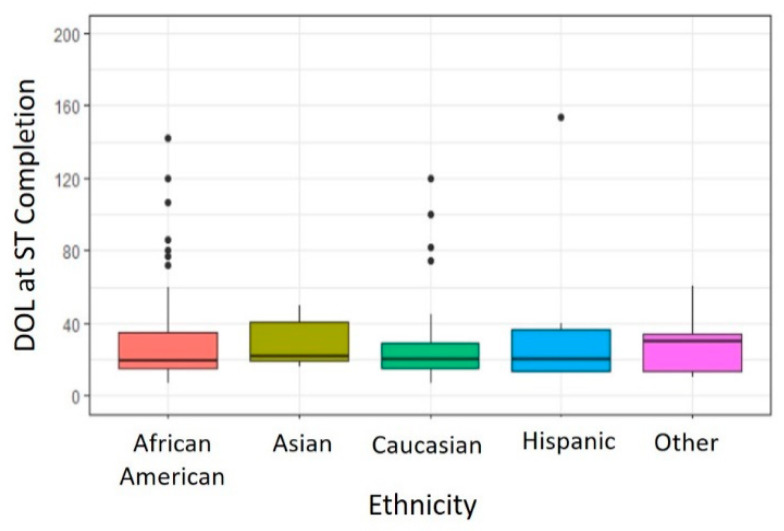
Effect of Ethnicity on Age at Successful Completion of Sweat Chloride Testing.

There were no significant differences in DOL at ST completion noted between different ethnicities (*p =* 0.874). As shown in Figure 3, infants identified as Caucasian (Median 20 days, Range 7–120 days, *n* = 73 infants) completed ST on a DOL similar to those infants identified as African American (Median 19.5 days, Range 7–142 days, *n* = 58 infants), Asian (Median 22 days, Range 16–50 days, *n* = 5 infants), Hispanic (Median 20.5 days, Range 13–154 days, *n* = 6 infants) or Other (Median 30 days, Range 10–61 days, *n =* 13 infants) ethnicity.

#### 3.1.4. Gestational Age

Preterm infants (<37 weeks gestation) demonstrated later DOL at ST completion (Median 35 days, Range 12–142 days, *n =* 19 infants) compared with term (≥37 weeks gestation) infants (Median 18.5 days, Range 7–150 days, *n =* 132 infants) (*p =* 0.001). For preterm infants, the median gestational age was 35 weeks and average gestational age was 34.2 ± 2.7 weeks. Range in preterm infant gestational age was 27–36 weeks. The overall QNS rate for initial ST in preterm infants was 50% (*n =* 9/18 infants) and 17.4% in term infants (*n* = 23/132 infants).

#### 3.1.5. Skin Lesions

None of the patients referred for ST had skin lesions noted which delayed ST completion.

### 3.2. Laboratory Factors

**Figure 4:**
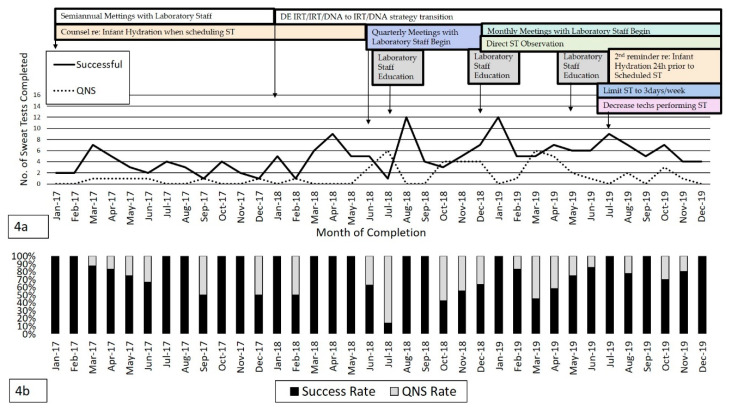
(**a**) Total monthly number of successful and ‘quantity not sufficient’ sweat chloride test results from January 2017–December 2019. Timing of quality improvement measures (as outlined in Table 1) are included for correlation with sweat chloride test quantity not sufficient (QNS) and success numbers and rates. (**b**) Monthly percentages of successful and ‘quantity not sufficient’ sweat chloride results from January 2017–December 2019.

ST is shown in Figure 4a which represents the total number of successful and QNS tests per month from January 2017 to December 2019 as QI measures were instituted and adjusted. Over 2 years, an average of ~6.2 ST/month were completed with 4.9 ST/month successful on the first attempt and 1.4 ST/month resulting as QNS. The average success rate was 78% (*n =* 175/224 tests completed). Figure 4b demonstrates the ST success and QNS percentages over the study period. A rise in QNS rate between June–August 2018 was noted which subsequently improved following institution of QI measures as shown in Figure 4a. However, there was a subsequent rise in QNS rates again between October-December 2018 likely due to changes in testing behaviors or decreased adherence with QI processes in place. Examples included: inappropriate placement of Macroduct disc, aggressive cleaning site and collection discs, and timing of sweat collection and large volume of technicians completing testing (4–5 providers). Overall, ST QNS rates remained ≤10% following additional QI initiatives added in July 2019 which included direct observation of ST completion (shown in Supplementary Materials), and limitation of ST to 3 days/week with reduced technicians completing testing (2 providers) in order to maintain proficiency. The rise in QNS rate in October 2019 represented 1 patient in whom ST was difficult to complete due to likely dehydration and delayed recovery following a minor medical procedure.

## 4. Discussion

This QI study illustrates efforts to improve the timeliness of successful ST completion at our CFF-accredited Center. ST QNS rates vary across the United States [20]. Delays in ST contribute not only to postponing CF diagnosis and initiation of therapy but also contribute to parental stress and anxiety while awaiting ST results [23] and may lead to additional expensive testing. While the number of ST completed at our Delaware CF Center is low compared with larger, more densely populated regions of the United States, it is still important to systematically assess factors that may impact completion of diagnostic ST completion in a timely manner. The lower number of tests also allows more individualized analysis of infant and laboratory factors which may contribute to QNS rates and may be generalized to other Centers routinely addressing their ST success and QNS rates.

Sweat production has been assessed on DOL 1 in infants as young as 36 weeks gestational age and by ~DOL 13 in infants <36 weeks [24]. Current CFF guidelines suggest ST may be completed within the first 2 weeks of life (but greater than 24 h of age) in infants with BW >2.0 kg [16]. Despite these suggestions, many States have documented QNS rates >10% despite guidelines provided by the CFF [25,26,27]. Over 2 years, the average ST QNS rate at our CF Center was 22% but fluctuated depending on interventions and decreased to an average of 14% in the last 6 months of the study when addressing infant and laboratory factors. Overall, the number of ST completed monthly for infants was low which also may affect QNS rates due to decreased numbers of testing opportunities/month for technicians to maintain proficiency.

Our study confirms that BW and gestational age are important factors in timing of ST completion. While previous studies suggest infant BW >2.0 kg is sufficient for ST completion [28], our fluctuating QNS rates led us to stratify ST completion based on varying infant BW: <2.0 kg, 2.0–3.0 kg, 3.0–3.5 kg and >3.5 kg as 3.0 kg is the minimum BW recommendation made by the United Kingdom for ST [29]. Additionally, in the United States, Collins et al. demonstrated that BW >3.0 kg was associated with decreased QNS ST results [30]. In our patients, infants with BW >3.0 kg consistently completed ST at an earlier age with sufficient sweat volume compared with those BW <3.0 kg. While assessing the impact of measures which might improve ST QNS rates, infants were scheduled for ST after a weight of ~3.0 kg was achieved, particularly in 2019. Additionally, preterm birth has been associated with increased ST QNS rates [27,28,30]. Kleyn et al. suggested increased odds of QNS ST results in preterm gestation and low BW infants [27]. In the current QI study, infants with preterm gestation exhibited increased QNS rate and successfully completed ST at later DOL compared with infants born at full-term. While awaiting ST completion based on infant size or gestational age, our CF nurse coordinator remained in close communication with infant PCPs and provided education to families to ensure that the infant was thriving and not demonstrating any other symptoms or signs suggestive of CF while awaiting diagnostic test results. Psychosocial support for family member was available if needed.

All ethnicities were adequately represented in our QI population. Historically, Thomson et al. did not report any difference in density or distribution of eccrine sweat glands in European adults compared with African adults [31]. Additionally, sweat electrolyte analyses by di Sant’Agnese and colleagues presumed no differences in sweat glands based on racial background [32]. Much like Abdulhamid et al., which addressed ST QNS rates across CF Centers in Michigan [25], in our QI population, there was no difference in time to successful ST completion between Caucasian and African American or other ethnicities unlike other prior reports [13,28].

A rigorous QI model was utilized to address laboratory factors affecting ST outcomes. Regular and in-service education provided by clinical laboratory staff and MSC manufacturer improved ST QNS rates. Closer monitoring and direct observation further improved technical performance. In keeping with CFF recommendations, particularly in a small State with a lower number of abnormal NBS referrals, the number of clinical laboratory specialists completing ST was decreased in order to maintain proficiency [20]. Similarly, ST completion was limited to 3 days/week. Fluctuations in ST success/QNS rates highlight the importance of ongoing QI measures between the CF center and clinical laboratory staff to sustain best practices and investigate areas for future improvement [26].

## 5. Conclusions

In our CF NBS clinic, BW >3.0 kg and gestational age <37 weeks were found to significantly affect DOL at ST completion. No other infant demographic factors (sex, ethnic background, skin lesions) were noted to affect DOL at ST completion. Laboratory personnel require ongoing education and feedback to maintain proficiency in ST. Smaller centers and testing sites may have difficulties meeting recommendations for completing 1–2 tests/week and outliers will affect QNS rates to a greater degree in these locations. QI measures are useful to assess factors which may contribute to ST QNS rates at a CF center and strategize processes to improve and sustain ST success rates.

## Figures and Tables

**Table 1:** Quality Improvement Initiatives to Improve Sweat Testing Quantity Not Sufficient Rates.

Scheduled quarterly meetings with clinical laboratory staff/leadership. Laboratory education by CF Center Staff, Manufacturer. Scheduled monthly meetings with clinical laboratory staff/leadership. Laboratory direct supervision of sweat testing. Reduction in the number of laboratory staff completing sweat testing. Limiting sweat testing to 3 days/week. Duplicate pre-sweat test appointment phone calls by CF Center and Laboratory Staff. Identifying infant factors contributing to decreased sweat yield.

**Table 2:** Referral State of Infants with Abnormal Cystic Fibrosis (CF) Newborn Screening and Their Outcomes.

Infants Evaluated	2017–2019
Total Referred	160 infants
Referral State	
Delaware	69.3% (*n =* 111 infants)
Pennsylvania	26.3% (*n* = 42 infants)
New Jersey	4.4% (*n =* 7 infants)
Outcomes	
Normal (Non-CF)	86.9% (*n* = 139 infants)
CF	6.2% (*n* = 10 infants)
CRMS/CFSPID	6.9% (*n* = 11 infants)

## Supplementary Materials

The following are available online at https://www.mdpi.com/2409-515X/7/1/1/s1.
