# Supplementary material for: Newborn Screening for Cystic Fibrosis: Infant and Laboratory Factors Affecting Successful Sweat Test Completion
Source: Int J Neonatal Screen. 2020 Dec 25;7(1):1. doi: 10.3390/ijns7010001 (PMC7838990; doi:10.3390/ijns7010001)
Supplement: Supplementary file 1 [file IJNS-07-00001-s001.pdf]

Nemours. Alfred I. duPont Hospital for Children

Department of Pathology/Clinical Laboratory
1600 Rockland Rd
Wilmington DE 19899

2020 SWEAT TEST COLLECTION TRAINING/ASSESSMENT

POSITION: PHLEBOTOMIST NAME: ______________________________ DATE: ______________

OBSERVER: ______________________

SWEAT TESTING: Successful completion of Sweat test training/assessment. It is required for sign off on phlebotomy training for sweat test procedure. For each category indicated, the phlebotomist must demonstrate acceptable performance by random monthly observation/s.

Random Assessments/Observations- one per month (video viewed once annually or if education recommended or desired)
Annual Comp Assessments/Observations- for each associate that performs sweat testing –video is mandatory

RANDOM MONTHLY ASSESSMENT ☐ VIDEO N/A ☐ Y ☐

LINK TO MACRODUCT Sweat Test Collection Video = Wescor Macroduct Usage

INSTRUMENTATION

☑**Check Box When Performed**

SWEAT TEST PROCEDURE

Patient Testing Check Off

- CLEAN/PREP SITE ☐
- ELECTRODE STIMULATION WITH PILOCARPINE ☐
- MICRODUCT COLLECTION DEVICE PLACEMENT ☐
- MICRODUCT COLLECTION AFTER 30 MINUTES ☐
- MICRODUCT SWEAT LABELING ☐
- MACRODUCT VIDEO VIEWED (link provided) ☐

DATE AND INITIAL AT COMPLETION OF ASSESSMENT

Collector Initial: ________ Date: __________ Observer Initial: _______ Date: __________

Supervisor Initial: ________ Date:__________
